# Use of Mesenchymal Cells to Modulate Immune Suppression and Immune Reconstruction in a Patient with Aplastic Anemia Complicated by Invasive Sino-Orbital Aspergillosis

**DOI:** 10.4274/tjh.2013.0041

**Published:** 2014-06-10

**Authors:** Hakan Özdoğu, Mahmut Yeral, Can Boğa, İlknur Kozanoğlu

**Affiliations:** 1 Başkent University Adana Adult Bone Marrow Transplantation and Stem Cell Therapy Center, Department of Hematology, Adana, Turkey; 2 Başkent University Adana Adult Bone Marrow Transplantation and Stem Cell Therapy Center, Department of Physiology, Ankara, Turkey

**Keywords:** Mesenchymal stem cell, Immune recovery, Aplastic anemia, allogeneic stem cell transplantation

## Abstract

Cultured human bone marrow mesenchymal cells (MSCs) have immunomodulatory and tissue regenerative properties. This report summarizes the result of post-transplant treatment with MSCs of a 26-year-old patient with aplastic anemia complicated by invasive sino-orbital aspergillosis. The patient was treated with MSCs to benefit from the dual effects of MSCs in immune reconstitution: suppression against alloreactive T cells and facilitation of the re-engraftment process. The patient did not develop acute or chronic graft-versus-host disease. The aspergillus infection healed completely. The engraftment failure was also ended without any complications. During his last visit in his fourth year after transplantation, the patient was in hematological remission. Human bone marrow-derived MSCs seem to have an important role in preventing or overcoming immunological complications in patients who undergo stem cell transplantation.

## Abstract (tr)

Kemik iliği kaynaklı mezenkimal kök hücrelerin (MKH) immun düzenleyici ve doku tamir edici özellikleri vardır. Bu yazıda komplike invaziv sino-orbital aspergillozu olan 26 yaşındaki aplastik anemili olguda post transplant MKH uygulanması rapor edilmiştir. MKH ile tedavi edilen hastada MKH’lerin immun yeniden yapılanma üzerine çift yönlü etkisinden faydalanılarak; alloreaktif T hücreleri suprese edilmesi ve yamalanmanın kolaylaştırılması amaçlanmıştır. Komplike hastada akut ve kronik graft versus host hastalığı gelişmedi. Aspergilloz enfeksiyonu ve engraftmant yetmezliği komplikasyonsuz düzeldi. Takip eden visitlerinde hasta dördüncü yılında hematolojik remisyondadır. Kök hücre transplantasyonu yapılan hastalarda insan kemik iliği kaynaklı MKH’lerin uygulanması immunolojik komplikasyonlardan korunma ve önlenmesinde önemli role sahiptir.

## INTRODUCTION

The infectious complications and immune dysfunction after human hematopoietic stem cell transplantation (HSCT) can activate acute graft-versus-host disease (GvHD) among patients undergoing HSCT [1]. The restoration of immune function is critical in effective treatment of invasive fungal infection and prevention of acute GvHD in these patients [2]. Mesenchymal stem cells (MSCs) are capable of regulating immune function and supporting marrow stroma [1,2,3,4,5]. 

Here we present a patient with very severe aplastic anemia and invasive aspergillosis who was successfully treated with hemopoietic stem cells from a sibling donor and MSCs from original and third-party donors.

## CASE PRESENTATIONS

A 26-year-old male patient with aplastic anemia was admitted to our center with fever, periorbital swelling, periorbital pain, and bloody nasal discharge. The patient had been diagnosed with severe aplastic anemia 10 days before, when the bone marrow examination (aspiration and biopsy) was compatible with severe idiopathic aplastic anemia (bone marrow cellularity of <10%). Empirical ceftazidime initiated for infection control was ineffective. Nasal and right periorbital erythema had developed 3 days before admission. On physical examination, fever of 38.5 °C, nasal and right periorbital erythema, and right periorbital swelling were detected. Ecchymosis and petechial lesions over the lower limbs were also present. Examination of the eyes was normal and no neurological abnormalities were detected. His hematological test revealed pancytopenia (WBC: 0.33x10^9^/L, Hb:7 g/L, Plt:19x10^9^/L). The percentages of lymphocytes, neutrophils, and eosinophils in the peripheral blood were 91%, 1.9%, and 2.4% of total nuclear cells, respectively. The percentage of reticulocytes in the peripheral blood was 0.13% of erythrocytes. Peripheral blood smear revealed normochromic and normocytic erythrocytes and decreased leukocytes (mostly lymphocytes) and platelets. Serologic tests for antibodies to hepatitis B and C virus, cytomegalovirus, Epstein-Barr virus, and human immunodeficiency viruses 1 and 2; serological tests for antinuclear antibodies; and a direct Coombs’ test were all negative. Biochemical tests revealed normal vitamin B12, folic acid, and serum iron. Computed tomography of the paranasal sinuses demonstrated a space-occupying lesion of the right maxillary and ethmoid sinus region. Histopathology showed numerous PAS-stained Aspergillus hyphae without tissue invasion. Tissue culture revealed Aspergillus fumigatus.

The patient was a student. At the time of admission, he reported that he had not used any drugs or been exposed to any known chemicals recently. 

Voriconazole administration was started with limited clinical benefit (12 mg/kg/day). However, 20 days later, the patient developed fever and flare of nasal discharge. 

**The First Transplant**

A human leukocyte antigen (HLA)-compatible sibling bone marrow donor was available for transplantation. Hence, the patient underwent non-myeloablative allogeneic peripheral HSCT with a conditioning regimen including antithymocyte globulin (5 mg/kg/day for 3 days) and cyclophosphamide (50 mg/kg/day for 4 days). Five million cells per kilogram of CD34 cells were infused from the HLA-matched sibling donor. Prophylaxis against GvHD was given with cyclosporine and methotrexate. Naturally, the patient was at high risk of GvHD because of the active fungal infection. For use prior to the possible onset of acute GvHD, human bone marrow MSCs, cultured ex vivo, were obtained from his original donor under good manufacturing practice conditions (Thanks to Prof. Dr. Ercüment Ovalı).

Following peripheral HSCT, neutrophil engraftment occurred on day +16. However, platelet recovery was not complete until day +32. Culture-expanded MSCs were infused on day +32 to the patient for hematopoietic support and their immunosuppressive effect to prevent acute GvHD. The MSC dose used was 1x106/kg. Platelets engrafted within 10 days following MSC infusion. On day +60, complete peripheral blood count revealed the following: Hb, 8 g/L; WBC, 2.2x10^9^/L (30% neutrophils, 68% lymphocytes, 2% monocytes); Plt, 70x10^9^/L. A chimerism study revealed 100% donor T-cell chimerism in the peripheral blood. He had no cytopenia or evidence of acute GvHD on days +90 and +180. No early or late adverse events were demonstrated after MSC infusion. There was no improvement of the patient’s fungal infection after MSC infusion.

**Second Transplant**

The patient developed late donor-type engraftment failure in the eighth month. The use of immunosuppressive therapy including corticosteroids and cyclosporine for control of engraftment failure was ineffective. The patient therefore underwent a second transplant from the original donor using the same conditioning protocol. Human MSCs were obtained from an HLA-mismatched unrelated donor. This time, to promote engraftment, MSCs were given 4 h before stem cell infusion. The doses of MSCs and CD34 cells were 0.8x106/kg and 6.43x10^6^/kg, respectively. No lymphocyte depletion was performed because of absence of GvHD. Times to reach an absolute neutrophil count greater than 0.5x10^9^/L and a platelet count greater than 50x10^9^/L were 7 days and 9 days, respectively. Graft function was normal on day +100 of the second transplant. No early or late adverse events were demonstrated after MSC infusions. Informed consent was obtained from the patient for the applied procedures. Approval of the MSC infusion was also received from the National Regulatory Authorities. We are now following this patient as an outpatient in the post-transplant fourth year.

## DISCUSSION

Acute GvHD and graft failure/graft rejection remain the main clinical challenges in allogeneic HSCT. As an important risk factor, active infections may influence the probability of incidence and severity of GvHD [1]. Post-transplant immunosuppressive therapy (mostly cyclosporine A and methotrexate) for prevention of GvHD may not be effective, especially in high-risk patients [1]. In addition, delayed engraftment or graft rejection may also be associated with severe infections [6]. McCann et al. [7] reported the poor outcome of graft failure patients (actuarial survival of 17%), where infection was an important contributory factor.

Human bone marrow MSCs are pluripotent cells that are able to home to damaged tissues and differentiate into various cell lineages such as adipocytes, fibrocytes, neural cells, and osteocytes [5]. They may also support the growth of bone marrow cells [3,4,5]. Several reports have shown the suppressive effects of MSCs on T cells, B cells, dendritic cells, and natural killer cell proliferation in vitro and in vivo [5]. For this reason, there has recently been great interest in the use of MSCs for the treatment of acute GvHD, but there is very little knowledge on prevention of GvHD with the use of MSCs [8,9,10,11]. In recent studies, MSCs have been shown to exert beneficial effects on hemopoietic recovery, prevention of graft rejection, and prevention or control of GvHD following allogeneic HSCT [12,13]. 

Fast engraftment was vital for our patient with active infection while he was undergoing transplantation. Thus, we decided to culture MSCs from the original donor, although we were not able to co-infuse MSCs with stem cells due to technical reasons during the first HSCT. We did MSC infusion on day +32 in order to at least prevent acute GVHD. Engraftment occurred as we wanted, with no GVHD, and the patient recovered quickly.

A second marrow transplant is the treatment of choice for aplastic anemia patients with no engraftment or graft rejection unresponsive to immunosuppressive therapy after HSCT [7]. We observed severe destruction of engrafted bone marrow cells, unresponsive to filgrastim and efficient immunosuppressive treatment, in the eighth month. Therefore, we decided to give the patient a second HSCT. To accelerate engraftment of the second allograft, co-infusion of a second dose of MSCs was planned [8]. This time, to prevent delay in infusion of MSCs, bone marrow MSCs from an HLA-mismatched unrelated donor were used based on the literature knowledge on this issue [9,10]. Successful and sustained engraftment occurred without any complications. 

Although a definitive conclusion cannot be made for MSCs’ usage, we think that MSCs allowed us to obtain a positive outcome from the point of view of the literature knowledge as discussed below. It has been reported that MSC infusion together with allogeneic hematopoietic stem cells resulted in fast engraftment of neutrophils and platelets [11]. Further support for the enhancement of hematopoietic recovery was presented by Le Blanc et al. [8]. They showed that MSCs promoted donor cell engraftment in patients with primary or secondary graft failure. It was also reported that MSC treatment suppressed acute GvHD in transplanted patients, who had better overall survival than patients in the control group [12]. Other investigators supported this observation [9,10]. 

MSCs seem to be a promising treatment option for stem cell recipients who need fast hematopoietic recovery owing to co-morbidity. 

## CONFLICT OF INTEREST STATEMENT

The authors of this paper have no conflicts of interest, including specific financial interests, relationships, and/ or affiliations relevant to the subject matter or materials included. 
